# Attitudes Toward and Susceptibility to Doping in Spanish Elite and National-Standard Track and Field Athletes: An Examination of the Sport Drug Control Model

**DOI:** 10.3389/fpsyg.2021.679001

**Published:** 2021-06-08

**Authors:** Elena García-Grimau, Ricardo De la Vega, Rafael De Arce, Arturo Casado

**Affiliations:** ^1^Department of Physical Education, Sport and Human Movement, Universidad Autónoma de Madrid, Madrid, Spain; ^2^Department of Digital Communications, Spanish Agency for Health Protection in Sport, Madrid, Spain; ^3^Department of Applied Economics, Universidad Autónoma de Madrid, Madrid, Spain; ^4^Centre for Sport Studies, Rey Juan Carlos University, Madrid, Spain; ^5^Faculty of Health Sciences, Isabel I University, Burgos, Spain

**Keywords:** attitudes, behavior, doping, morality, track and field athletes, performance-enhancing drugs, sport drug control model

## Abstract

The Sport Drug Control Model (SDCM) is likely to be the model which most explicitly represents the theoretical paradigm of the psychological study of the use of doping in sport. This model can be further developed through its analysis in different populations and cultures. The main aim of this study was to empirically test the SDCM while analyzing for the first time the intentions and attitudes toward doping in Spanish track and field athletes. A secondary aim was to assess the extent to which the variables in the model together predict attitude, susceptibility, and behavior toward the use of performance-enhancing substances. Participants were 281 Spanish elite and national-standard track and field athletes from whom 80.1% were 18-28 years old and 49.5% were females. Participants completed the SDCM questionnaire measuring morality, legitimacy, benefits appraisal, threat appraisal, self-efficacy to refrain from doping, reference groups' endorsement of doping methods/substances, use of legal supplements, availability and affordability of doping, attitudes toward doping, susceptibility to doping and, self-reported use of banned performance-enhancing substances or methods. Structural equation modeling supported a good fitness of the SDCM and confirmed that positive attitudes toward doping predicted high susceptibility to doping (β = 0.55, *p* < 0.001), which is in turn associated with the use of prohibited substances and methods (β = 0.12, *p* < 0.05). The factors that have most influence on attitudes toward doping are morality (β = 0.46, *p* < 0.001) and reference group opinion (β =0.62, *p* <0.001). Self-reported doping use was 9.6%. These findings confirm SDCM reproducibility and variability (as it accounts for several variables) in Spanish track and field competitive athletes. It is recommended to implement preventive programs which allow athletes to acquire a strong moral stance against doping and coaches to employ the tools required to instill and educate their athletes in rejecting these illegal practices that corrupt the integrity of competitive sport.

## Introduction

Social science research in doping in sport attempts to understand why athletes dope and how they do it; hence it helps to improve educational and interventional anti-doping programs. Whereas, investigation on biomedical and legislative aspects of doping began in the 1960s (Beckett and Cowan, 1978), research on psychosocial doping factors was initiated in the 1990s (García-Grimau et al., 2020). Understanding the psychology of doping remains a challenge for social researchers due to the complex nature of the different variables influencing doping behavior (Blank et al., 2016). Researchers in this field have explored through different theoretical models all the possible factors that influence intentions and attitudes toward doping behavior (Donovan et al., 2002; Strelan and Boeckmann, 2003; Petróczi and Aidman, 2008).

The Theory of Planned Behavior (TPB, Ajzen, 1991) has been widely utilized to understand the psychological mechanisms underpinning the use of doping in sport (Barkoukis et al., 2013). The TPB is based on the principle that personal intentions to perform a certain behavior are the strongest predictor of that behavior. These intentions are in turn determined by three other factors: attitudes, subjective norms, and the control of perceived behavior (Armitage and Conner, 2001). Different integrative models that incorporate the TPB as a cornerstone have been developed (Strelan and Boeckmann, 2003; Petróczi and Aidman, 2008). One of them is the Sport Drug Control Model (SDCM).

The SDCM incorporates different frameworks from the behavioral sciences (Nicholls et al., 2014) and takes into account the particularities of competitive sport, such as the existence of legal methods to improve performance and the influence of environmental and cultural beliefs (Lazuras, 2016). The World Anti-Doping Agency (WADA) provides a Social Science Research Package with a useful guideline for measuring athletes' responses in each of the SDCM domains that influence doping attitudes and behavior (World Anti-Doping Agency, 2015). The authors who developed the SDCM (Donovan et al., 2002) propose that attitudes and intentions to dope are influenced by six factors: morality (whether doping and cheating is right or wrong), legitimacy (how athletes perceive anti-doping organizations to have strong authority to enforce anti-doping regulations), benefits appraisal (beliefs about the benefits of doping), threat appraisal (beliefs about the negative consequences of doping), personality (personality traits or psychological factors) and reference group opinions (subjective norms, social approval of doping) along with two “market factors”: availability/trafficking and affordability of doping method or substances. These attitudes and intentions are in turn strong predictors of doping behavior. The SDCM has been quantitatively examined twice in Australian elite athletes and the items used have shown validity and reliability (Gucciardi et al., 2010; Jalleh et al., 2013). Jalleh et al. (2013) reported that morality, reference group opinion and legitimacy are significantly associated with doping attitudes. Nicholls et al. (2020) developed an adaptation of the SDCM aimed at adolescent athletes and found that morality construct was the strongest predictor of attitudes toward doping in this population. The SDCM is likely to be the model that most explicitly represents the theoretical paradigm of the psychological study of the use of doping in sport (Kirby et al., 2016). This model was extended to consider other factors like the use of legal supplement and technologies, and broader social and cultural contexts. However, it is necessary to analyze the model in different populations and cultures to further develop it (Jalleh et al., 2013).

Moreover, studies on attitudes and behavior toward doping in Spanish athletes are scarce and mainly focused on cycling (Morente-Sánchez et al., 2013a,b), soccer (Horcajo and de la Vega, 2014, 2016; Morente-Sánchez and Zabala, 2015; Horcajo and Luttrell, 2016) and triathlon (Morente-Sánchez et al., 2013a; Maestre, 2015). However, among all summer Olympic sports the greatest numbers (i.e., 205, 6 and 295) of athletes' anti-doping rule violations worldwide in 2016, in the Spanish context in 2016, and adverse analytical findings in 2017, respectively, were reported in athletics (World Anti-Doping Agency, 2018, 2020).

Hence, the main aim of this study was to empirically test the SDCM while analyzing for the first time the intentions and attitudes toward doping in Spanish elite and national-standard track and field athletes; and to assess the extent to which the variables in the model together predict attitude, susceptibility, and behavior toward the use of performance-enhancing substances (PES).

## Materials and Methods

### Participants and Design

A cross-sectional online survey was conducted to examine Spanish track and field (athletics) athletes in 2020. A total number of 339 athletes accepted the consent form and 289 athletes completed the survey, from whom eight were excluded due to the existence of non-responses in most of the items measuring dependent variables (i.e., doping behavior, attitudes, and susceptibility to doping), leaving a final sample of 281 participants. Participants were selected according to their performance level. The inclusion criterion was having achieved a qualification standard for participation in either a senior or age category (under 20 years [U-20] or under 23 years [U-23]) national athletics championship. Twenty-four training groups nationwide were contacted through their respective coaches. WhatsApp 2.18.52 (Mountain View, California, USA) groups were created, and athletes were invited to participate in the online survey (via link). Coaches and athletes were informed about the aims and objectives of the study. In the first section of the online survey athletes received information explaining the objectives and procedures of the study and consent to take part. Participants were reassured about the anonymity and confidentiality of their responses and about their right to withdraw at any time.

The sample was composed of both male and female (i.e., 50.5 and 49.5% of the sample, respectively) athletes. Most of the participants (i.e., 80.1% of the sample) were aged between 18 and 28 years. Regarding the level of performance of the sample, 5.6, 18.2, 14.7, 10.1, 44.8, and 6.8% of the subjects had participated at least once in Olympic Games, World Athletics Championships, European Athletics Championships, other international events with the national team, national Athletics Championships, and regional Championships, respectively. Three athletes failed to report their level of performance. Regarding the athletic discipline, 61.6, 4.3, 17.1, 13.9, and 3.2% of the athletes mainly participated in middle- and long-distance running, race walking, sprinting/hurdle, jumping/throwing, and combined events, respectively.

### Instrument

WADA's questionnaire package was used to measure the following constructs in the SDCM: (1) morality; (2) legitimacy; (3) benefits appraisal; (4) threat appraisal; (5) personality traits; (6) beliefs about reference groups' endorsement of doping methods/substances; (7) use of legal supplements; (8) beliefs about the availability of PES and relevant authorities' control over trafficking of doping methods/substances; (9) beliefs about the affordability of doping methods/substances; (10) attitudes toward doping, (11) susceptibility to doping; and (12) self-reported use of banned PES or methods.

All the items in the questionnaire belong to the Social Science Research Package (World Anti-Doping Agency, 2015) with the exception of moral disengagement which was measured using the 6-items from Moral Disengagement in Doping Scale (Kavussanu et al., 2016). Moral disengagement is a cognitive mechanism theorized by Bandura (1991) that has been strongly correlated with doping attitudes and intentions toward doping (Kavussanu et al., 2019; Stanger and Backhouse, 2020).

Moreover, Donovan et al. (2002) did not include doping susceptibility in the SDCM as a predictor of doping behavior, however susceptibility to doping has been reported to be a strong predictor of doping behavior linked to attitudes toward doping (Gucciardi et al., 2010; Barkoukis et al., 2013; Blank et al., 2016; Nicholls et al., 2020). For this reason, this construct has been included as a dependent variable in our analysis, along with doping attitudes and behavior.

The final 44-items questionnaire covers all the modules described in section five of WADA's social science package (Table 1 and Appendix 1). The questionnaire was translated into Spanish by experts in the field of anti-doping, sport science and sport psychology, then a sworn translation was carried out.

**Table 1:** Description of the questionnaire.

**Modules/constructs from WADA guidelines**	Construct measures^*^	Question number^**^
Morality	Moral decision-making, moral stance, moral affect, moral disengagement (6-items scale, Kavussanu et al., 2016).	Q1, Q2, Q3, Q4
Legitimacy	Distributive justice.	Q5, Q6, Q7
Benefit appraisal	Perceived performance-enhancing effects of banned substances and methods, Likelihood of potential positive outcomes.	Q8, Q9, Q10, Q11
Threat appraisal	Threats of enforcement, threats relating to ill-health effects.	Q12, Q13, Q14, Q15
Personality traits	Self-efficacy to refrain from doping, goal orientations.	Q16, Q17
Reference Groups' endorsement of doping methods/substances	Subjective norms.	Q18, Q19, Q20
Availability of PESM and relevant authorities' control over trafficking of doping methods/substances	Perceived availability of PES, access to banned PES, perceived access to medical advice on use of PES, perceived efforts of relevant authorities in enforcing laws against trafficking of PESM.	Q21, Q22, Q23, Q24, Q25
Affordability of PESM	Perceived affordability of PESM.	Q26
Beliefs about other athletes' attitudes toward and use of doping	Descriptive norms.	Q27
Belief about societal influences on doping	Belief about societal influences on doping.	Q28, Q29, Q30
Use of nutritional supplements and other technologies	Use of nutritional supplements, use of other technologies	Q21, Q32
Performance-enhancing drug use	Frequency of use of PESM in the past 12 months.	Q33. Q34
Demographic and sporting background	Athletics discipline, competition level, income from sport, age group, and gender.	Q35, Q36, Q37, Q38, Q39
Overall Susceptibility to doping	Susceptibility, attitudes, and intention to doping.	Q40, Q41, Q42, Q43, Q44

* *See section Limitations and Future Research of WADA's social science research package for full descriptions of measures and items*.

** *See Appendix 1 in the Supplementary Material*.

*WADA, World Anti-doping Agency; NADO, National Anti-doping Organization; PES, performance-enhancing substances; PESM: performance-enhancing substances and methods*.

### Protocol

Ethics committees from Isabel I de Castilla International University (UI1-PI016) and World Anti-Doping Agency (2019-A2) provided ethical approval for the completion of the present study. All of the participants signed a consent form to participate in this study which was conducted in accordance with the Declaration of Helsinki. Athletes were informed about the aims and purposes of the study and reassured about their anonymity and confidentiality of their data.

### Data Analysis

The latent variables used in the statistical analysis to test the SDCM are shown in Table 2. Descriptive and internal reliability analysis of the study variables was performed using Statistical Package for the Social Sciences (SPSS) version 24.0 (IBM, Armonk, NY, USA). We calculated the means (95% CI), standard deviations (SDs), McDonald's ω, composite reliability (CR) and average variance extracted (AVE) values as a measure of reliability and internal consistency. Results are shown in Table 2. The range of percentage of missing values for each indicator variable was relatively low (i.e., 0.4-3.1%), they were assumed to be missing at random and, when necessary, they were imputed using the expectation maximization method (Graham, 2009).

**Table 2:** Variables, descriptive statistics and internal reliability estimates for the variables measuring the sport drug control model through structural equation modeling.

**Latent variables**	**Indicator variables**	**Range**	**Mean**	**SD**	**ω**	**CR**	**AVE**
Doping behavior	Self-reported use of PES and prohibited methods.^†^	(0) Never use to (1) ever use	0.09	0.29	–	–	–
Susceptibility to doping	Consideration of an offer to use PES	(1) not at all to (4) a lot of consideration	1.35	0.51	0.82	0.88	0.72
Attitudes toward doping	Perceived necessity to use PES^*^	(1) definitely don't have to use to (4) definitely have to use	2.04	1.10	–	–	–
Morality	Moral disengagement.	(1) Strongly disagree to (7) strongly agree	1.51	0.77	0.72	0.84	0.57
Legitimacy	Distributive justice^†^	(1) Very secure to (5) Not at all secure	2.62	0.82	0.91	0.92	0.76
Benefit appraisal	Incentives for performing well^*^	(1) not at all to (3) a lot	2.42	0.43	0.77	0.85	0.65
Threat appraisal	Deterrence in and out of competition^†^	(1) Very likely to (5) Not at all likely	3.09	1.26	–	–	–
	Threat to health^†^^*^	(1) A lot of harm to (5) no harm	2.56	0.92	0.90	0.92	0.66
Personality traits	Self-efficacy to refrain from doping.^*^	(1) completely capable to (7) Not at all capable	1.74	1.51	0.96	0.96	0.88
Reference Groups' Endorsement of Doping Methods/Substances	Subjective norms^*^	(1) Probably disapprove to (5) would definitely approve	1.33	0.42	0.78	0.87	0.70
Use of nutritional supplements	Frequency of nutritional supplements use^†^	(1) Never to (4) systematically	2.65	0.89	0.70	0.78	0.54

*PES, performance-enhancing substances; PESM, performance-enhancing substances and methods; SD, standard deviation; ω, McDonald's ω; CR, composite reliability; AVE, average variance extracted*.

† *Transformed Variable*.

* *Reverted scale*.

To test the SDCM (Jalleh et al., 2013), structural equation model (SEM) was carried out using AMOS package for SPSS version 24.0. We made an examination of the measurement portion of the model and setting constraints to avoid identification issues. To evaluate the adequacy of the model we employed the fit indices recommended in guidelines (Marsh et al., 2005; Tabachnick and Fidell, 2013): ratio of the χ^2^ to the degrees of freedom (χ^2^/df <2), comparative fit index (CFI ≥ 0.95), Tucker Lewis Index (TLI ≥ 0.95), root-mean-square error of approximation (RMSEA ≤ 0.08) and Standardized Root-Mean-Square Residual (SRMR ≤ 0.10).

## Results

**Figure 1:**
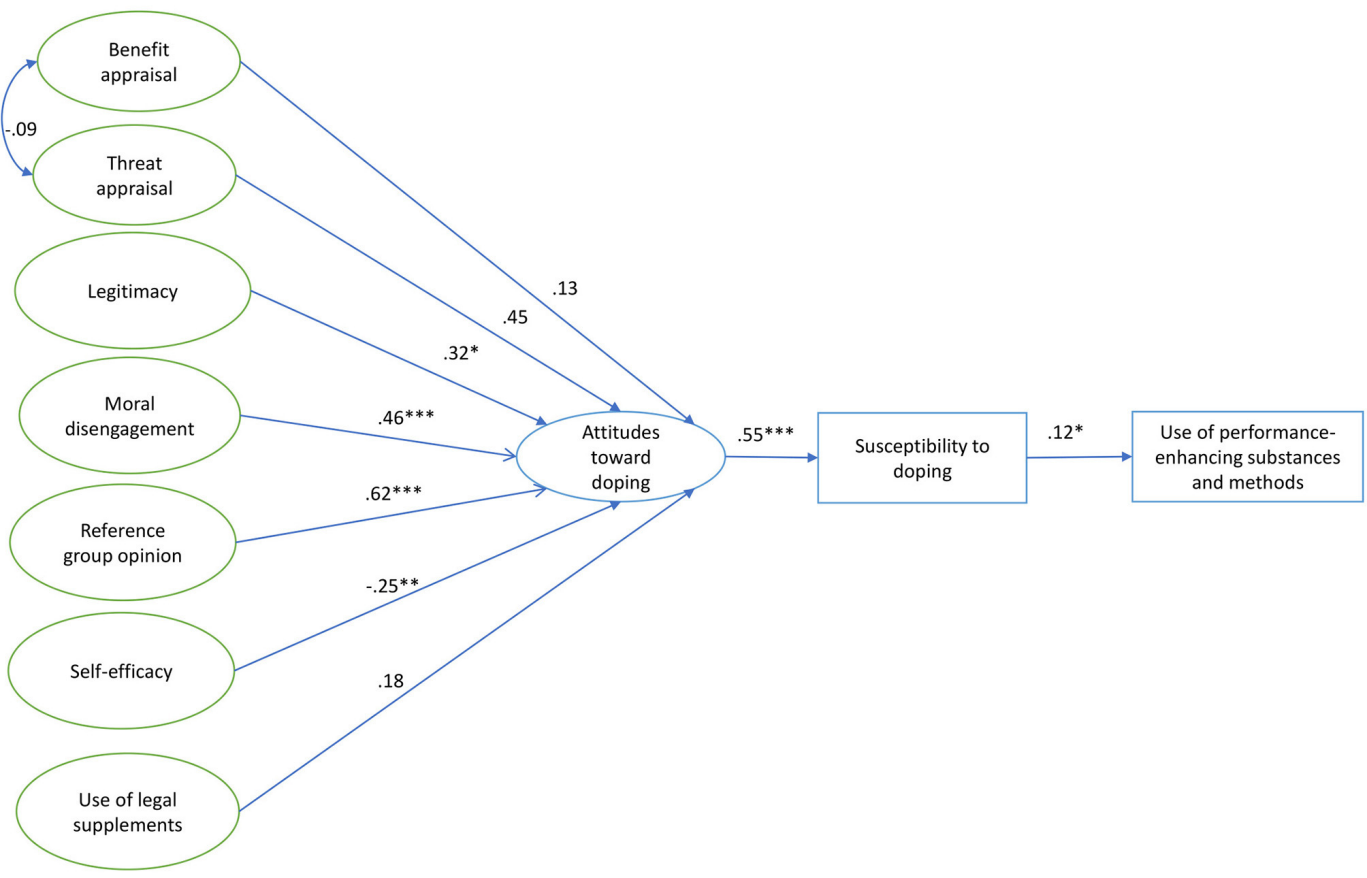
Overview of results of structural equation model analysis with standardized parameter estimates. Different levels of significance according to *p*-value: *p < 0.05, **p < 0.01, and ***p < 0.001.

The market factors availability and affordability could not be considered in the model due to a high percentage of “I do not know” responses. Regarding doping prevalence, 9.6% of the sample self-reported use of prohibited substances or method, from whom 3.2 and 6.4% of the sample self-reported the use of PES and prohibited methods, respectively. Descriptive and internal reliability of study variables are shown in Table 2. Omega (ω) and CR values are >0.7 and AVE values are >0.5, which indicates a good reliability and internal consistency of the questionnaire suitable for SEM analysis. The SEM analysis of the SDCM revealed a good fit of the data (see Figure 1): χ^2^/df = 1.46, *p* < 0.001, CFI = 0.96, TLI = 0.96, RMSEA = 0.041 (90% CI = 0.032, 0.049), SRMR = 0.08. Covariance between benefit and threat appraisal did not change the model fitness and improved the standardized parameter estimates and significance. Standardized parameter estimates are reported in Figure 1, which show that attitudes toward doping is a significant predictor of doping susceptibility (β = 0.55, *p* < 0.001) which is in turn a significant but lesser predictor of doping behavior (β = 0.12, *p* < 0.05). Reference group opinion (β = 0.62, *p* < 0.001) and morality (β = 0.46, *p* < 0.001) are the strongest and most positive predictors of attitudes toward doping. Personality (β = 0.25, *p* < 0.01) and legitimacy (β = 0.32, *p* < 0.05) showed a significant but moderate relationship with attitudes toward doping. Threat appraisal approached significance (β = 0.32, *p* = 0.056) and the rest of the latent variables (benefit appraisal and supplement use) were not significantly related (*p* > 0.1).

## Discussion

Doping attitudes and behaviors were analyzed for the first time in Spanish elite and national-standard track and field athletes. The percentage of self-reported doping (prohibited method and substances) (i.e., 9.6% of the sample) is similar to that found in other studies have measured doping prevalence through questionnaires in elite athletes from other countries belonging to individual and team sports. Self-reported lifetime prevalence in those studies ranges from 4 to 10% (García-Grimau et al., 2020). However, it should be noted that only the use of prohibited substances and not the use of prohibited methods were reported (Barkoukis et al., 2013; Jalleh et al., 2013; Al Ghobain et al., 2016; Kim and Kim, 2017). Nonetheless, self-reported doping prevalence data remain greater than the prevalence resulting from analytical measurements of doping control samples. In this regard, the frequency of adverse analytical findings in individual and team sports reported by WADA from 2014 to 2017 was 1.0% (Aguilar-Navarro et al., 2020).

To the best of the authors' knowledge by the time of writing, the SDCM was examined in Australian athletes (Jalleh et al., 2013) and adolescent athletes (SDCM-AA) from United Kingdom, Australia, United States, and Hong Kong (Nicholls et al., 2020). Therefore, it was suitable to test the model's applicability in other countries and populations of athletes (Jalleh et al., 2013) and thus the examination of the SDCM in Spanish elite- and national-standard track and field athletes is a strength of our study. Present results displayed that positives attitudes toward doping predicted high susceptibility to doping, which is in turn associated with the use of prohibited substances and methods. The observed strength of the relationship between attitudes toward doping and doping susceptibility is in agreement with results from other studies (Gucciardi et al., 2010; Barkoukis et al., 2013; Blank et al., 2016; Nicholls et al., 2020). Nevertheless, assuming the theoretical principles of psychological models that investigate doping in sport and due to the difficulty of measuring doping behavior, assessing athletes' attitudes toward doping or susceptibility to doping may be sufficient to better understand the psychological mechanisms underpinning doping behavior (Kirby et al., 2016). Despite the bias of social desirability in self-reporting prohibited behavior, we were able to analyze the direct and significant relationship between susceptibility and doping behavior.

The factors that were found to have the greatest influence on attitudes toward doping are morality and reference group opinion. In previous analysis using the SDCM and SDCM-AA, morality was found to be one of the factors that has most influence on attitudes toward doping (Jalleh et al., 2013; Nicholls et al., 2020). Similarly, recent literature reviews showed that moral variables are strong predictors of doping attitudes and behaviors (Ntoumanis et al., 2014; Backhouse et al., 2015). In the present study, morality was measured under the concept of moral disengagement, using the scale of Moral Disengagement of Doping in Sport (MDDS, Kavussanu et al., 2016). Bandura's theory 1991 proposed that there are a number of moral disengagement mechanisms to justify a transgressive act that violates moral standards and thus minimize negative effect and protect self-esteem. In this way, the higher score in the MDDS, the more favorable attitudes toward doping. In recent studies sampling competitive athletes from different countries, moral disengagement was found to be a strong predictor of positive attitudes toward the use of PES (Hodge et al., 2013; Kavussanu et al., 2019; Ring and Hurst, 2019). Moreover, other social, environmental and personal factors may influence the use of doping through their effects on moral disengagement (Kavussanu et al., 2016). Present results emphasize the potential effectiveness of introducing the concept of morality into anti-doping educational programs through practical interventions in order to acquire a strong moral stance against doping and avoid morality disengagement in athletes who are highly susceptible to dope. We emphasize the importance of further implementing the concept of morality in anti-doping education. Intervention programs oriented toward changes in the moral aspect of doping have displayed the greatest effectiveness in antidoping prevention and they are scarcely implemented unfortunately (Gatterer et al., 2020).

Reference group opinion was found to be a significant and positive predictor of attitudes toward the use of doping, which means that the greater endorsement of doping by athletes' reference groups (i.e., coach, teammates, and family) the more prone were attitudes toward doping. This finding is consistent with that from Jalleh et al. (2013). In addition, these results are also in agreement with those from Lazuras et al. (2010) regarding subjective norms, which is a variable that derives from the TPB. The role played by significant others is a crucial contextual variable in understanding attitudes toward doping in athletes. If athletes' closest entourage rejects doping, this would be a protective factor to prevent athletes from being tempted to use banned substances or methods. Athletes are highly influenced by their reference group, mainly by their coaches, but surprisingly there are just a few studies carried out in this population (Backhouse et al., 2015). In general, coaches display negative attitudes toward doping but feel inadequately trained to engage in anti-doping actions (Mazanov et al., 2014; Moston et al., 2014; Backhouse et al., 2015; Morente-Sánchez and Zabala, 2015). Athletes' support personnel may need to be highly involved in anti-doping education and receive a specific training. They should not only transmit passive information, but also foster its role as the main barrier to doping and learn how to translate the knowledge acquired into practice, through a preventive education based on the intervention.

There was a significant and moderate relationship between attitude toward doping and both personality traits and legitimacy. Personality traits were measured using the self-efficacy to refrain from doping scale (Lucidi et al., 2008). In a recent meta-analytic review, self-efficacy to refrain from doping displayed the strongest negative correlation with doping intentions and behaviors (Ntoumanis et al., 2014) which means that the less ability to avoid doping or resist temptations, the more positive attitudes toward it were observed.

In line with Jalleh et al. (2013) results, benefit and threat appraisal did not reach significance. This means that in our study sample the potential benefits or positive outcomes that an athlete could achieve by cheating, are not a significant factor influencing positive attitudes toward doping. Regarding threat appraisal, a low level of threat perceived by the athlete due to deterrence effect or risk to health, does not predict more positive doping attitudes, despite this factor is being close to significance (*p* = 0.056). In our sample, the degree of perceived threat is generally moderate. Threat appraisal could be significant if it would be measured in athletes who either have cheated or have already had a first contact with doping.

The SDCM was published by Donovan et al. (2002), who did not propose the use of sport supplements yet. Afterwards, a review by World Anti-Doping Agency (2015) led to an expansion of the model and more domains were included such as the use of legal supplements and technologies. In the present study this novel variable which was not previously examined by either Jalleh et al. (2013) or Gucciardi et al. (2010) was included. Current literature has reported that athletes typically consume legal diet supplements (Baltazar-Martins et al., 2019) and some studies have associated the use of diet supplements with positive attitudes toward doping (Ntoumanis et al., 2014; Hurst et al., 2020). However, our results show that the use of diet supplements is a weak predictor of attitudes toward doping in comparison with other factors like morality, reference group opinion or personality traits.

Overall, the existence of many factors and different models to analyze doping attitudes and behaviors is evident, which makes the study of the psychosocial phenomenon of doping complex. This is translated into real life in a variety of possible situations of temptation in athletes that can lead them to engage in doping practice. Effective and active prevention is needed, not just the receipt of passive information in terms of anti-doping education.

## Limitations and Future Research

Availability and affordability indicators of the different latent variables evaluated were not included in the SEM due to a high number of “I do not know” responses reported in these specific questions (57.1 and 46.6%, respectively). Moreover, due to the limitations of structural equation modeling, some factors and items included in the questionnaire could not be analyzed. SEMs require a series of mathematical restrictions in terms of the number of equations and observed and latent variables. These restrictions require a balance for their identifiability, and it is always necessary to respond to this mathematical conditioning by including, if necessary, more variables than those recommended by the principle of statistical parsimony (Tarka, 2018). Further studies with a deeper examination of the data, variables, and with a different statistical approach, may provide valuable information for a better understanding of the complex phenomenon of doping. Additionally, the prevalence data of the study does not differentiate the athletes who reported having used prohibited PESs without permission from those who also did so while being authorized to use PES/PESs through a therapeutic use exemption (TUE) due to having reported a specific illness or condition which requires the use of a certain medicine. On the other hand, only one (i.e., use of PES) from the 11 WADA current doping-related infractions (World Anti-Doping Agency, 2021) was evaluated. Furthermore, bias derived from the methodology used and social desirability in self-reported drugs and the rest of prior limitations suggest that the real doping prevalence outcome found in the present study might be underestimated. In order to be able to assess a real doping prevalence outcome, the use of indirect measures such as randomized responses or fuzzy models are recommended. These measures avoid the existence of the aforementioned limitations and have reported greater doping-related prevalence outcomes than those used in the present study and previous ones evaluating the SDCM (Pitsch et al., 2007; Ulrich et al., 2018; García-Grimau et al., 2020). There is a wide variety of psychological factors which influence attitudes toward doping. Researchers have examined some personality variables altogether. Self-efficacy to refrain from doping, sport motivation (Ring and Kavussanu, 2018), goal orientation (Hardwick et al., 2021), dark triad personality traits (Matosic et al., 2016; Nicholls et al., 2019) and perfectionism (Madigan et al., 2020) have shown a strong relationship with attitudes toward and susceptibility to the use of doping. However, in the present study only “self-efficacy to refrain from doping” was analyzed. Therefore, further studies examining other psychological variables and their influence on attitudes toward doping are encouraged. In addition, the specific reasons for which diet supplements are consumed by athletes might be of greater influence on attitudes toward doping than either frequency of consumption or the types of supplements used and therefore the former should be also studied. In addition, a recent study (Hurst et al., 2020) has shown that the use of some types of sport supplements can influence to a greater extent on attitudes toward doping than other types. Therefore, as the type of supplements use was not accounted for in the present study, the extent to which this variable influenced on attitudes toward doping might have not been very precisely analyzed. Accordingly, further studies should also ask the specific type of sport supplement used in order to more accurately predict attitudes toward doping. Moreover, further research is required to analyze the relationship between the socioeconomic context of the athletes and their attitudes toward doping.

## Conclusion

Overall, the SDCM displayed reproducibility and variability, having been tested through several ways using different constructs and variables, in the first analysis to date of attitudes toward the use of doping and doping-related behaviors in Spanish track and field competitive athletes. This is the first questionnaire written in Spanish analyzing the influence of several factors on doping attitudes and behaviors in athletes. The development of this questionnaire represents an important step forward for the antidoping community in order to be able to analyze and evaluate the attitudes and behaviors toward doping in a much wider population given the great number of Spanish speaking athletes worldwide. Morality and reference group opinion are the factors which have most influence on attitudes toward doping in these athletes. It is recommended to implement preventive programs, beyond the passive reception of information, which allow athletes to acquire a strong moral stance against doping and coaches to employ the tools required to instill and educate their athletes in rejecting these illegal practices that corrupt the integrity of competitive sport.

## Data Availability Statement

Data can not be available for ethical reasons according to an explicit condition set by the ethics committee from the World Antidoping Agency (2019-A2).

## Ethics Statement

The studies involving human participants were reviewed and approved by Ethics committees from Isabel I de Castilla International University (UI1-PI016) and World Antidoping Agency (2019-A2). The patients/participants provided their written informed consent to participate in this study.

## Author Contributions

EG-G designed, conceptualized, performed analysis, and wrote the paper. EG-G and AC collected the data. RaD contributed performing the statistical analysis. AC and RiD contributed to the writing. All authors contributed to the article and approved the submitted version.

## Conflict of Interest

The authors declare that the research was conducted in the absence of any commercial or financial relationships that could be construed as a potential conflict of interest.
