# Supplementary material for: Attitudes Toward and Susceptibility to Doping in Spanish Elite and National-Standard Track and Field Athletes: An Examination of the Sport Drug Control Model
Source: Front Psychol. 2021 Jun 8;12:679001. doi: 10.3389/fpsyg.2021.679001 (PMC8219072; doi:10.3389/fpsyg.2021.679001)
Supplement: Supplementary file 1 [file Data_Sheet_1.PDF]

Supplementary Material

Appendix 1. Sport Drug Control Model 44-items Questionnaire.

**Questions**	**Scale**
Q1. In the list below there are some things that people have said about cheating and fair play in sport. Please read each one and circle one of the numbers beside it to show how much you agree or disagree with it. Some of these are not very different so you will have to carefully examine each statement.<br><br>1. It is ok to cheat if nobody knows.<br>2. Winning and losing are a part of life<br>3. I sometimes try to wind up the opposition<br>4. I would cheat if I thought it would help me win.<br>5. It is ok to lose sometimes because in life you don't win everything.<br>6. It is not against the rules to psych people out so it's ok to do so.<br>7. If other people are cheating, I think I can too.<br>8. If you win properly, it feels better than if you did it dishonestly.<br>9. Sometimes I waste time to unsettle the opposition.<br>10. I cheat if I can get away with it.<br>11. You have to think about the other people and not just winning.<br>12. If I don't want another person to do well then I put them off a bit.<br>13. When I get the chance, I fool the official.<br>14. I get annoyed by people trying to "win at all Costs".<br>15. It is a good idea to upset your opponent.<br>16. I always play by the rules.<br>17. Winning is all that matters.<br>18. I would never psych anybody out.<br>19. I would cheat if I thought it would help me or my team win.<br>20. It is understandable that players swear in the heat of the moment.	(1) Strongly disagree to (5) Strongly agree
Q2. Regardless of whether you believe performance enhancing substances or methods (PESM) should be banned or allowed, which of the following statements best describes your own personal feelings about deliberately using banned PESM?<br><br>1. I believe deliberately using banned PESM to improve performance is morally wrong under any circumstances.	(1) to (3)

2. I believe deliberately using banned PESM to improve performance is morally OKunder some circumstances, but wrong under others.<br>3. I believe deliberately using banned PESM to improve performance is morally OK under any circumstances.	
Q3 If you were caught using banned performance enhancing substances or methods, to what extent would you experience the following feelings.<br><br>1. Ashamed<br>2. Embarrassed<br>3. Guilty	(1) Not at all to (5) a great extent
Q4. A number of statements describing thoughts that athletes might have about competitive sport are listed below. Please read these statements carefully and indicate your level of agreement.<br><br>1. Doping is alright because it helps your team.<br>2. Doping is just a way to "maximize your potential".<br>3. Compared to the illegal things people do in everyday life, doping in sport is not very serious.<br>4. Players cannot be blamed for doping if their teammates pressure them to do it.<br>5. A player should not be blamed for doping if everyone on the team is doing it.<br>6. Doping does not really hurt anyone.	(1) Strongly disagree to (7) strongly agree
Q5. How fair is AEPSAD in terms of treating all athletes equally?	(1) Very fair to (4) very unfair + I do not know
Q6. How secure is the AEPSAD'S drug testing procedures in Spain? That is, in the taking of samples and the care of samples?	(1) Very secure to (4) not at all secure + I do not know
Q7. How accurate do you feel the current drug tests are in terms of being able to correctly identify the following substances?<br><br>1. Anabolic steroids<br>2. Beta-blockers<br>3. Designer steroids like tetrahydrogestrinone (THG)<br>4. Erythropoietin (EPO) and other similar substances<br>5. Human growth hormones (hGH)<br>6. Diuretics	(1) Very accurate to (4) not at all accurate + I do not know

Q8. If you were to use the following substances, how likely is it that these substances would improve your performance in your sport?<br><br>1. Anabolic steroids.<br>2. Beta-blockers.<br>3. Designer steroids like tetrahydrogestrinone.<br>4. Erythropoietin (EPO) and other similar substances.<br>5. Human growth hormones (hGH).	(1) Definitely would not to (5) Definitely would + I do not know
Q9. If you were to use a banned performance enhancing substance of your choice, how likely is it that you would improve your performance in your sport?	(1) Definitely would not to (5) Definitely would + I do not know
Q10. How much would you personally like these outcomes for performing well in your sport?<br><br>1. National celebrity status<br>2. Lucrative financial sponsorship deals<br>3. Personal best achievements<br>4. Opportunities for remaining in the sport as coach, trainer or administrator<br>5. Future financial security<br>6. International celebrity status	(1) A lot to (3) not at all
Q11. To what extent does your sport offer athletes these outcomes if they perform well?<br><br>1. National celebrity status<br>2. Lucrative financial sponsorship deals<br>3. Personal best achievements<br>4. Opportunities for remaining in the sport as coach, trainer or administrator<br>5. Future financial security<br>6. International celebrity status	(1) A lot to (3) not at all
Q12. How likely is it that athletes at your level would be drug tested at least once a year?<br><br>1. In competition at least once a year.<br>2. Out of competition at least once a year.	(1) Very likely to (5) not at all likely + I do not know
Q13. It has been said that athletes who take banned substances can use various methods to avoid testing positive.	(1) Very likely to (5) not at all likely + I do not know

1. From what you know or have heard, if you were to take banned performance-enhancing substances while competing, how likely do you think that you could get away with it if you really tried to?<br>2. From what you know or have heard, if you were to take banned performance-enhancing substances out of competition, how likely do you think that you could get away with it if you really tried to?	
Q14. From what you know or have heard, are the penalties for a positive drug test in your sport severe or lenient?	(1) Very severe to (4) very lenient + I do not know
Q15. How much harm to your health do you think would be caused by using each of the following substances for a short time say up to two months?<br><br>1. Anabolic steroids.<br>2. Beta-blockers.<br>3. Designer steroids like tetrahydrogestrinone.<br>4. Erythropoietin (EPO) and other similar substances.<br>5. Human growth hormones (hGH).<br>6. Diuretics	(1) No harm to (4) a lot of harm + I do not know
Q16. Here are several scenarios that you may have encountered or may come across during your career in sport. Please rate the extent to which you currently feel capable in being able to avoid or overcome these situations using the scale below.<br><br>1. To avoid using banned PEDMs before a competition even when you know you can get away with it.<br>2. To avoid using banned PEDMs in sport even when you feel down physically.<br>3. To resist the temptation to use banned PEDMs to improve your performance.<br>4. To resist the temptation to use banned PEDMs to have a body that others would admire, even when no-one would ever know.<br>5. To resist the temptation to use banned PEDMs to have a great appearance.<br>6. To avoid using banned PEDMs to have your body look as you would like.<br>7. To avoid using banned PEDMs to get results more quickly, even when no one would ever know.<br>8. To not use banned PEDMs, despite the pressure to do so from others.<br>9. To avoid using banned PEDMs to improve your performance in the sport you practice, even when you know that it will not have side-effects.	(1) Not at all capable to (7) completely capable

10. To avoid using banned PEDMs, even when most of those who practice your sport use them	
Q17. Success in sport can mean different things to different people. The statements in this section of the survey capture a variety of ways in which athletes define their sporting success. Please read these statements carefully and indicate your level of agreement with each one.<br><br>In sport, I feel most successful when I ….<br><br>1. I reach personal goals.<br>2. I show clear personal improvement.<br>3. I perform to the best of my ability.<br>4. I overcome difficulties.<br>5. I reach a goal.<br>6. I work hard.<br>7. I show other people I am the best.<br>8. I am the best.<br>9. I am clearly superior.<br>10. I outperform my opponents.<br>11. I beat other people.<br>12. I win.	(1) Strongly disagree to (5) Strongly agree
Q18. If you decided to use a banned performance enhancing substance, to what extent do you think each of the following people would approve or disapprove, or would not care either way if you did that?<br><br>1. Your coach.<br>2. Parents.<br>3. Team mates/training partners.<br>4. Team doctor.<br>5. Close friends.<br>6. Manager.	(1) Would definitely approve to (5) Definitely disapprove
Q19. If you were considering using a banned performance enhancing substance, how much would you take into account these people's opinions on whether you should or should not do so?<br><br>1. Your coach.<br>2. Parents.<br>3. Team mates/training partners.<br>4. Team doctor.<br>5. Close friends.<br>6. Manager.	(1) Would definitely take into account to (5) Definitely not take into account

Q20. The following statements are intended to provide an insight into your beliefs about other people's opinions towards the athlete using doping.<br><br>1.Most people I know would approve of me using prohibited substances to enhance my performance during his season.<br>2.People who are important to me would approve of me using prohibited substances to enhance my performance during this season.<br>3.Most people close to me expect me to use prohibited substances to enhance my performance during this season.	(1) Strongly disagree to (7) strongly agree
Q21. How easy or difficult would it be for you to get each of the following types of substances if you wanted to?<br><br>1. Anabolic steroids.<br>2. Beta-blockers.<br>3. Designer steroids like tetrahydrogestrinone.<br>4. Erythropoietin (EPO) and other similar substances.<br>5. Human growth hormones (hGH).<br>6. Diuretics	(1) Probably impossible to (5) very easy + I do not know
Q.22 If you wanted to get and use a banned PES, which of the following people, if any, do you think would help you if you asked them to do so?<br><br>1. Your coach.<br>2. Parents.<br>3. Team mates/training partners.<br>4. Team doctor.<br>5. Close friends.<br>6. Manager.	(1) Would definitely help me to (5) definitely would not help me + I do not know
Q23. If you wanted to use a banned PES, how easy would it be to get good medical advice on how to use the substance?	(1) Probably impossible to (5) very easy + I do not know
Q24. How serious do you feel the following authorities are in preventing trafficking of banned performance enhancing substances in Spain?<br><br>1. Police/Guardia Civil<br>2. AEPSAD	(1) Not at all serious to (5) very serious

Q25. Overall, how effective do you feel the following authorities are in preventing trafficking of banned performance enhancing substances in Spain?<br><br>1. Police/Guardia Civil<br>2. AEPSAD	(1) Not at all effective to (5) very effective
Q26. How expensive would it be for you personally to buy each of the following types of substances?<br><br>1. Anabolic steroids.<br>2. Beta-blockers.<br>3. Designer steroids like tetrahydrogestrinone.<br>4. Erythropoietin (EPO) and other similar substances.<br>5. Human growth hormones (hGH).<br>6. Diuretics	(1) Very cheap to (5) very expensive + I do not know
Q27. The following statements are intended to provide an insight into your beliefs regarding other athletes' use of doping.<br><br>1. Out of 100%, how many athletes in your sport do you believe engage in doping to enhance their performance?<br>2. Out of 100%, how many elite athletes in your country do you believe engage in doping to enhance their performance?<br>3. Out of 100%, how many elite athletes do you believe will be engaged in doping during the next 2 years to enhance their performance?<br>4. Out of 100%, how many coaches in your sport do you believe would encourage their athletes to use doping to enhance their performance?<br>5. Out of 100%, how many coaches in elite sports in your country do you believe would encourage their athletes to use doping to enhance their performance?	Indicate percentage:
Q28. How much pressure, directly or indirectly, do you think the Spanish government or the Spanish Olympic Committee puts on elite athletes to win Olympic gold medals?	(1) No pressure at all to (4) a lot of pressure
Q29. To what extent, if at all, do you think commercial influences on the Olympics and sport in general have increased a 'win at all costs' attitude amongst elite athletes?	(1) Had no effect to (4) increase a lot
Q30. To what extent, if at all, do you think commercial influences on the Olympics and sport in general have increased the temptation amongst elite athletes to use banned performance enhancing substances?	(1) Had no effect to (4) increase a lot

Q31. How often have you used any of these nutritional supplements in the past 12 months?<br><br>1. Vitamin or mineral supplements.<br>2. Herbal products.<br>3. Creatine.<br>4. Sports drinks.<br>5. Energy bars.<br>6. Caffeine.<br>7. Protein-carbohydrate shakes.	(1) Never to (6) Systematically
Q32. How often have you used any of these methods or technologies in the past 12 months?<br><br>1. High altitude stages.<br>2. Hypoxic training.<br>3. Intravenous therapy with permitted substances.<br>4. Ozone therapy	(1) Never to (6) Systematically
Q33. Which one of the following most applies to you?<br><br>1. I have never considered using a banned performance-enhancing substance.<br>2. At one stage I thought briefly about using a banned performance-enhancing substance.<br>3. At one stage I thought quite a bit about using a banned performance-enhancing substance.<br>4. I still think occasionally about using a banned performance-enhancing substance because other athletes are using them.<br>5. I briefly used a banned performance-enhancing substance in the past but no longer do so.<br>6. I occasionally use a banned performance-enhancing substance now for specific purposes.<br>7. I regularly try or use banned performance-enhancing substances.	(1) to (7)
Q34. In the last 12 months, how often have you used any of the following, for whatever reason?<br><br>1. Anabolic steroids.<br>2. Beta-blockers.<br>3. Designer steroids like tetrahydrogestrinone.<br>4. Erythropoietin (EPO) and other similar substances.<br>5. Human growth hormones (hGH).<br>6. Diuretics	(1) Had never use to (6) more than 10 times

7. Doping methods	
Q35. Indicate the athletic discipline you practice:<br><br>1. Sprinting – hurdles<br>2. Middle distance / long distance (includes race walking on road).<br>3. Jumps and throws<br>4. Multi-events	(1) to (4)
Q36. What is the highest level you have competed at?<br><br>1. Olympic games<br>2. World championship<br>3. European championships<br>4. Other International competition with the national team<br>5. National level competition<br>6. Regional level competition	(1) to (6)
Q37. What is your total annual income from all sport sources?<br><br>1. Less than 5,000 euros.<br>2. 5,000 to 9,999.<br>3. 10,000 to 19,999.<br>4. 20,000 to 39,999.<br>5. 40,000 to 59,999.<br>6. More than 60,000	(1) to (6)
Q38. What is your age group?<br><br>18-20<br><br>21-24<br><br>25-28<br><br>29-32<br><br>33-36<br><br>More than 37	(1) to (6)
Q39. Are you:<br><br>Male<br><br>Female	(1) to (2)

Q40. If you were offered a banned PES under medical supervision at low or no financial cost and the banned performance enhancing substance could make a significant difference to your performance and was currently not detectable, how much consideration do you think you might give to this offer?	(1) None at all to (4) a lot of consideration
Q41. Given the pressures athletes are often under to win, how confident are you that you could refuse this offer?	(1) Very confident could refuse to (5) would not want to refuse
Q42. How confident are you in being able to resist pressure from your team mates to use a banned substance?	(1) Very confident could resist to (5) would not want to resist
Q43. Do you intend to use prohibited substances or methods to enhance your performance or gain a competitive edge against your opponents during this season?	(1) Definitely not to (5) definitely will
Q44. In your sport, how necessary do you believe it is for athletes to use banned PES at least at some time, to perform at the very highest levels?	(1) Definitely have to use to (5) definitely do not have to use

AEPSAD: Agencia Española de Protección de la Salud en el Deporte (Spanish National Anti-doping Organization); PES: performance-enhancing substances; PESM: performance-enhancing substances and methods.
